# Development and Comparison of New Voltammetric Procedures for the Determination of In(III) Using ASV and AdSV Techniques with SBiµE as an Green Working Electrode

**DOI:** 10.3390/molecules30224377

**Published:** 2025-11-13

**Authors:** Malgorzata Grabarczyk, Wieslawa Cwikla-Bundyra

**Affiliations:** Department of Analytical Chemistry, Institute of Chemical Sciences, Faculty of Chemistry, Maria Curie-Sklodowska University, 20-031 Lublin, Poland

**Keywords:** indium(III), anodic stripping voltammetry, adsorptive stripping voltammetry, solid bismuth microelectrode, interference of organic substances

## Abstract

The article describes innovative procedures for determining In(III) using anodic stripping voltammetry (ASV) and adsorptive stripping voltammetry (AdSV) with cupferron as a chelating agent. In both procedures, an environmentally friendly solid bismuth microelectrode (SBiµE) with a diameter of 25 µm was used as the working electrode. In both procedures, 0.1 mol L^−1^ acetate buffer with a pH of 3.0 ± 0.05 was used as the supporting electrolyte. The electrochemical measurement conditions were as follows: −2.4 V for a 20 s activation step and −1.2 V for a 20 s accumulation step for ASV, and −2.5 V for a 45 s activation step and −0.65 V for a 10 s accumulation step for AdSV. The signal was recorded as a result of a positive potential change from −1.0 to −0.3 V in the case of the ASV procedure and as a result of a negative potential change from −0.4 to −1.0 V in the case of the AdSV procedure. The calibration graph was linear from 5 × 10^−9^ mol L^−1^ to 5 × 10^−7^ mol L^−1^ with a detection limit of 1.4 × 10^−9^ mol L^−1^ for ASV and from 1 × 10^−9^ mol L^−1^ to 1 × 10^−7^ mol L^−1^ with a detection limit of 3.9 × 10^−10^ mol L^−1^ for AdSV. The effect of interferents such as surfactants, humic substances and EDTA on the analytical signal was compared in the case of signal recording using the ASV technique with the signal recorded using the AdSV technique. Based on the results obtained, it was determined how the charge of interferents affects the signal depending on the technique used. To validate the practical application of the developed procedures, an analysis of In(III) recovery from samples of the Baltic Sea and Synthetic Sea Water was performed.

## 1. Introduction

In the European and global context, indium has been classified as a critical element, which means that its availability is crucial for the development of new technologies. The EU and the US are conducting monitoring programmes and raw material security strategies in which indium plays a strategic role alongside lithium, cobalt and neodymium. Indium plays a key role in the electronics industry, especially in semiconductor technology. Its compounds are used in the production of high-speed integrated circuits, semiconductor lasers, photodetectors and infrared devices. One of the most common and well-known uses of indium in the modern world is its presence in LCD (liquid-crystal display) and touch screens in smartphones, tablets, computer screens, etc. Due to its conductive and optical properties, indium is essential in the production of thin-film solar cells [1,2,3,4,5]. Most of the harmful effects on the human body result from occupational exposure of people working in factories where indium oxide is used. It can cause breathing problems leading to lung damage, as well as irritation and burns to the skin or eyes [6].

Widespread use of indium means that, together with industrial waste, it is entering the environment in increasingly large quantities in an uncontrolled manner. Therefore, the development of increasingly better and more sensitive procedures for the determination of indium in various environmental matrices is an extremely topical issue. For economic reasons, the low cost of determinations and their availability in analytical laboratories is also an important aspect. The methods that meet these requirements include, first and foremost, stripping voltammetry, which, due to its low equipment cost, is available in many analytical laboratories. Additionally, this method allows for low detection limits in many environmental matrices. It is therefore not surprising that this technique is very often used to determine trace amounts of many elements, mainly metal ions [7,8,9,10]. While dozens or even hundreds of voltammetric procedures dedicated to most metal ions are described in the literature, there are only a few for the determination of In(III). As early as 1962, a voltammetric procedure for determining In(III) using the techniques and advantages of anodic stripping voltammetry with linearly varying potential was described in the world literature [11]. The working electrode was a conventional mercury drop, and the proposed procedure allowed for the simultaneous determination of indium and tin. In a subsequent study, it was proven that indium exhibits completely different behaviour when determined by ASV on a hanging mercury electrode (HMDE) than on a thin-film electrode. Measurements carried out in various electrolytes showed that the thin film electrode was far more sensitive to the chemical form of indium in solution, and for maximum sensitivity, bromide or iodide had to be present in the supporting electrolyte [12]. Another study examined the effect of various surfactants on the anodic voltammetric peak of indium and other metals in terms of its size and potential. It was observed that in the presence of some surfactants, the indium peak decreases, while in the presence of others, a beneficial effect on its size was observed. All studies were conducted using a mercury drop as the working electrode [13]. Another publication based on the ASV technique and using HMDE focused on the determination of In(III) in the presence of Cd(II) and Pb(II) proposed conditions for conducting determinations that allow for the simultaneous determination of these three elements [14,15]. In addition to the ASV technique, the AdSV technique was also used for the determination of indium on a mercury electrode, using morin [16], ammonium pyrrolidine dithiocarbamate (APDC) [17], xylenol orange [18], 4-(2-pyridylazo)-resorcinol (PAR) [19] and cupferron [20] as complexing agents, respectively. In all these cases, indium was accumulated on the mercury electrode in the form of complexes, and the analytical signal was obtained as a result of their reduction. With growing awareness of the toxicity of mercury in recent years, research on indium analytics has focused on replacing harmful mercury electrodes with electrodes that are more environmentally friendly for the laboratory. To this end, Nafion-modified glassy carbon electrodes [21], antimony film electrodes [22,23], lead film electrodes [24] and bismuth film electrodes [15,19,25,26] have been proposed. As can be seen, most procedures were developed using bismuth film electrodes. This is consistent with the general direction of development of voltammetric procedures in recent years, in which bismuth electrodes account for an increasing share [27,28]. Bismuth film electrodes are undoubtedly more environmentally friendly than mercury electrodes, but their use requires the introduction of bismuth ions into the sample solution, from which a metallic bismuth film is generated. In order to eliminate the introduction of bismuth solution into the sample, and consequently into the waste after measurement, Pauliukaitė et al. introduced the bismuth bulk electrode as a new solid-state electrode. This electrode was a 4.0 mm diameter macroelectrode and was successfully used to determine metal ions using stripping voltammetry [29]. Gęca et al., in turn, proposed and described the preparation of a 25 µm diameter solid bismuth microelectrode (SBiµE), which allowed for additional advantages of the microelectrode over the macroelectrode, such as a favorable signal-to-noise ratio [30].

Thanks to this, there is no need to introduce bismuth ions into the sample solution, and to perform the measurement, it is sufficient to add only a buffer as the basic electrolyte to the sample solution. This electrode has been successfully used to determine metal ions such as thallium, achieving very low detection limits [31]. The aim of the research described in this paper is to use an environmentally friendly, green chemistry-compliant solid bismuth microelectrode to determine trace concentrations of In(III). Two techniques were used for the research: anodic stripping voltammetry and adsorption stripping voltammetry, thanks to which two independent procedures for the determination of In(III) were developed, allowing low detection limits to be obtained and enabling determinations to be carried out directly in environmental waters.

## 2. Results

### 2.1. Selection of the Supporting Electrolyte

The acetate buffer provides a suitable environment for the electrochemical determination of indium and is also the most commonly used medium for measurements with bismuth-based (micro) electrodes, regardless of whether they are configured as a bismuth layer or solid bismuth. Therefore, in our procedure, an acetate buffer was selected to provide a sufficiently stable environment ensuring an acidic pH; analogous measurements were also carried out using acetic acid as the base electrolyte. The tests were carried out for the acetate buffer in the pH range from 3 ± 0.05 to 4.6 ± 0.05. The highest analytical signal was obtained for pH = 3 ± 0.05 and was used as the base electrolyte in both ASV and AdSV measurements.

### 2.2. SBiµE Activation Step

#### 2.2.1. Potential of Activation SBiµE

**Figure 1:**
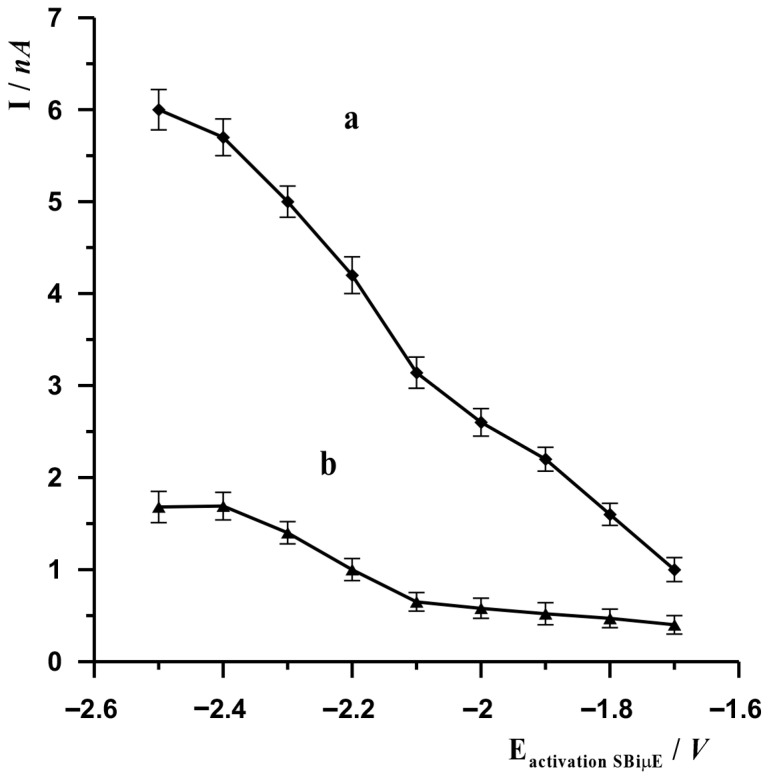
The influence of the SBiµE activation potential on the voltammetric signal of In(III) when performing measurements using the AdSV (a) and ASV techniques (b). AdSV measurement conditions: activation time 45 s, potential and accumulation time −0.65 V for 10 s. ASV measurement conditions: activation time 20 s, potential and accumulation time −1.2 V for 20 s. Solution composition 0.1 mol L^−1^ acetate buffer (pH = 3.0), 3 × 10^−8^ mol L^−1^ for ASV and 0.1 mol L^−1^ acetate buffer (pH = 3.0), 3 × 10^−8^ mol L^−1^, 7 × 10^−5^ mol L^−1^ cupferron for AdSV.

In the voltammetric procedure, the potential of the working electrode is an essential parameter ensuring the proper course of reactions occurring at the electrode. In the proposed procedure using a solid bismuth microelectrode, due to the electrode material used, it is necessary to activate the electrode surface before the actual stage of accumulation of the analyte being determined. This is due to the fact that, when in contact with air, a layer of bismuth oxide may form on metallic bismuth, which prevents the analyte from accessing the metallic bismuth during the accumulation stage. Therefore, each measurement begins with the electrochemical activation of the electrode, which is carried out by applying a sufficiently negative potential to it, as a result of which the bismuth oxide is reduced to its metallic form. The effect on the signal amplitude of the SBiµE activation potential was examined using both AdSV and ASV measurement techniques. The results are presented in Figure 1. Based on the results obtained, the most optimal activation potential was selected as −2.5 V for AdSV measurements and −2.4 V for ASV measurements.

#### 2.2.2. Time of Activation SBiµE

Another parameter affecting the peak value of the indium, closely related to the accumulation potential, is its duration. Therefore, experiments were conducted to select the optimal SBiµE activation times separately for each of the AdSV and ASV techniques used. Based on the results obtained, it was found that the maximum activation time allowing for the highest analytical signal is 45 s for AdSV measurements and 20 s for ASV measurements. Further extension of the activation times for both techniques does not allow for further increases in the analytical signal and even causes its gradual decrease.

### 2.3. Indium Accumulation Step on SBiµE

#### 2.3.1. The Potential of SBiµE in the Accumulation Step

**Figure 2:**
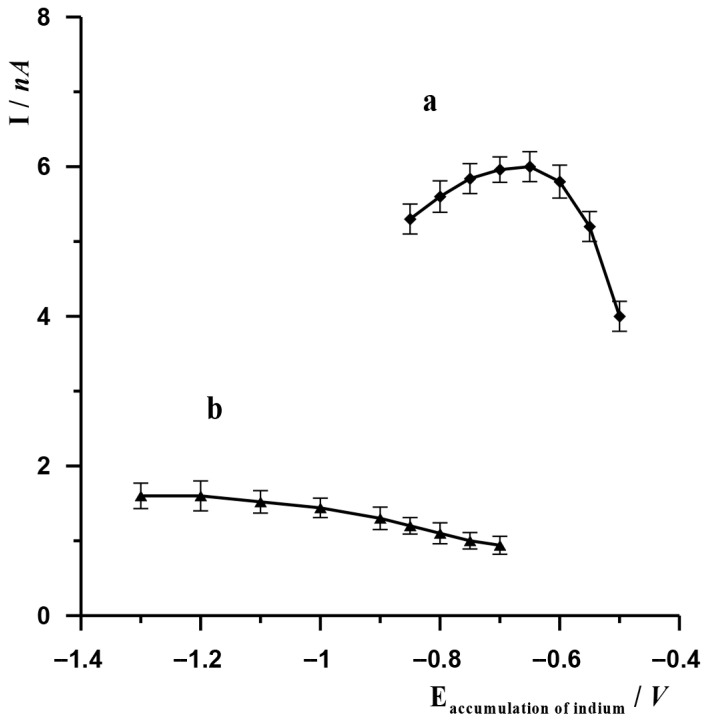
The effect of accumulation potential of In(III) on SBiµE on its voltammetric signal for AdSV (a) and ASV techniques (b). AdSV measurement conditions: activation potential and time −2.5 V and 10 s, accumulation time 10 s. ASV measurement conditions: activation potential and time −2.4 V and 20 s, accumulation time 20 s. Solution composition 0.1 mol L^−1^ acetate buffer (pH = 3.0), 3 × 10^−8^ mol L^−1^ for ASV and 0.1 mol L^−1^ acetate buffer (pH = 3.0), 3 × 10^−8^ mol L^−1^, 7 × 10^−5^ mol L^−1^ cupferron for AdSV.

Depending on the technique used, indium accumulation occurred based on various chemical reactions. In the case of the ASV technique, indium accumulation on SBiµE occurred as a result of the reduction of In(III) to metallic In(0), which ultimately occurred after optimisation at a potential of −1.2 V. The analytical signal was obtained as a result of oxidation of metallic indium, which in turn occurred as a result of a change in the potential in the positive direction. In order to select the accumulation potential, experiments were carried out in which, with the other measurement parameters remaining constant, only the accumulation potential was changed in the range from −1.3 V to −0.7 V. The other parameters remained unchanged: electrode potential and activation time −2.4 V and 20 s, accumulation time 20 s. Solution composition: 3 × 10^−8^ mol L^−1^ In(III), 0.1 mol L^−1^ acetate buffer. The results are presented in Figure 2 (graph b). As could be observed, at more negative accumulation potentials, the indium peak remained at a similar level, but as the accumulation potential changed towards less negative values, the indium peak gradually decreased. A potential of −1.2 V was selected as the most optimal for further measurements. In the case of the AdSV technique, the accumulation of indium on SBiµE occurred as a result of the adsorption of the In(III)-cupferron complex, which ultimately took place after optimisation at −0.65 V. The analytical signal was obtained as a result of the reduction of this complex, which occurred as a result of a change in potential in the negative direction. In order to select the accumulation potential, experiments were carried out in which, with the other measurement parameters remaining constant, only the accumulation potential was changed in the range from −0.85 V to −0.5 V. The other unchanged parameters were the electrode activation potential and time (−2.5 V and 45 s, respectively) and the accumulation time (10 s). The composition of the solution was 3 × 10^−8^ mol L^−1^ In(III), 7 × 10^−5^ cupferron, 0.1 mol L^−1^ acetate buffer. The results obtained are presented in Figure 2 (graph a). As observed, the highest indium signal was obtained using an accumulation potential of −0.65 V, at which the adsorption of the In(III)-cupferron complex on SBiµE occurs. At higher and lower values of this potential, the indium peak gradually decreases.

#### 2.3.2. Indium Accumulation Time on SBiµE

The accumulation time of indium on SBiµE is one of the key parameters affecting the size of the analytical peak, which directly affects the sensitivity of the determinations. In our procedures, the accumulation time in the ASV technique in the form of In(0) and in the AdSV technique in the form of In(III)-cupferron was tested using accumulation potentials of −1.2 V and −0.65 V, respectively. The composition of the solution was as follows: 3 × 10^−8^ mol L^−1^ In(III), 0.1 mol L^−1^ acetate buffer for ASV measurements, and an additional 7 × 10^−5^ mol L^−1^ cupferron was introduced in the AdSV technique. As observed in the case of measurements using both techniques, in the absence of an accumulation stage (0 s), we obtain an indium signal. This is due to the fact that during electrode activation, which occurs in the sample solution, indium also accumulates. However, as observed in both techniques, the introduction of the accumulation stage increases the indium signal, which determines the necessity of this step in obtaining the best possible sensitivity of the determinations. In the case of measurements performed using the AdSV technique, the most optimal accumulation time is 10 s, and its further extension causes a decrease in the indium signal. In the case of measurements performed using the ASV technique, it increases linearly with the extension of the accumulation time to 20 s, and then, with its further extension, the peak does not change.

### 2.4. The Influence of Cupferron in the AdSV Technique

In the AdSV technique, it is necessary to introduce a complexing agent into the solution, which will form a stable complex with the analysed analyte, in our case with In(III), which complex is electrochemically active and undergoes adsorption on the surface of the working electrode. One of the most commonly used complexing agents for In(III) determinations is cupferron, which was also used in our measurements. We investigated the effect of cupferron concentration on the indium signal in the concentration range from 10 to 100 µmol L^−1^. As observed, with an increase in cupferron concentration to 7 × 10^−5^ mol L^−1^, the indium peak increases, and at higher concentrations, it gradually decreases. Therefore, this cupferron concentration value was selected as optimal and was used as standard in the measurements.

### 2.5. Analytical Characterisation

#### 2.5.1. Procedure for Determining In(III) by the ASV Method

The relationship between the stripping peak current and concentration of In(III) was investigated under the optimal ASV conditions, i.e., supporting electrolyte 0.1 mol L^−1^ acetate buffer; activation of SBiµE potential and time −2.4 V and 20 s, respectively; accumulation on SBiµE potential and time −1.2 V and 20 s, respectively. The peak current recording on the voltammogram increased with the increasing concentration of In(III) in solution, with the dissolution peak potentials of indium around −0.6 V. The calibration curve exhibit excellent linear relationships with the peak currents and In(III) concentrations range from 5 × 10^−9^ mol L^−1^ to 5 × 10^−7^ mol L^−1^ and its equation was calculated as y = 0.055x − 0.128, where y is the anodic peak current (nA) and x is the concentration of In(III) in solution. In this case the linear correlation coefficient was r = 0.997. Furthermore, the limit of detection (LOD) was calculated from three-fold standard deviation and was equal to about 1.4 × 10^−9^ mol L^−1^ In(III).

The precision of the procedure was established by examining its repeatability and reproducibility. The repeatability of the measurements was evaluated from six subsequent measurement using different independent solutions containing 1 × 10^−8^ mol L^−1^ and 1 × 10^−7^ mol L^−1^ In(III). Relative standard deviations of the highest peak values were equal to 3.5% and 2.8%, respectively, for each concentration. The reproducibility was estimated from the measurements performed on six subsequent days and relative standard deviations was equal to 3.7% and 3.2% for 1 × 10^−8^ mol L^−1^ and 1 × 10^−7^ mol L^−1^ In(III), respectively.

#### 2.5.2. Procedure for Determining In(III) by the AdSV Method

The relationship between the stripping peak current and concentration of In(III) was investigated under the optimal AdSV conditions, i.e., supporting electrolyte 0.1 mol L^−1^ acetate buffer and 7 × 10^−5^ mol L^−1^ cupferron; activation of SBiµE potential and time −2.5 V and 45 s, respectively; accumulation on SBiµE potential and time −0.65 V and 10 s, respectively. The peak current recording on the voltammogram increased with the increasing concentration of In(III) in solution, with the reduction peak potentials of indium around −0.67 V. The calibration curve exhibit excellent linear relationships with the peak currents and In(III) concentrations range from 1 × 10^−9^ mol L^−1^ to 1 × 10^−7^ mol L^−1^ and its equation was calculated as y = 0.194x + 0.294, where y is the anodic peak current (nA) and x is the concentration of In(III) in solution. In this case the linear correlation coefficient was r = 0.996. Furthermore, the limit of detection (LOD) was calculated from three-fold standard deviation and was equal to about 3.9 × 10^−10^ mol L^−1^ In(III).

The precision of the procedure was established by examining its repeatability and reproducibility identically as described above in the ASV procedure. The repeatability and reproducibility were evaluated from solutions containing 5 × 10^−9^ mol L^−1^ and 5 × 10^−8^ mol L^−1^ In(III) and relative standard deviations was 3.5% and 3% for repeatability and 3.4% and 3.2% for reproducibility.

### 2.6. Comparison of the Influence of Organic Substances Present in the Sample Matrix on the Analytical Signal in the ASV and AdSV Procedures

**Figure 3:**
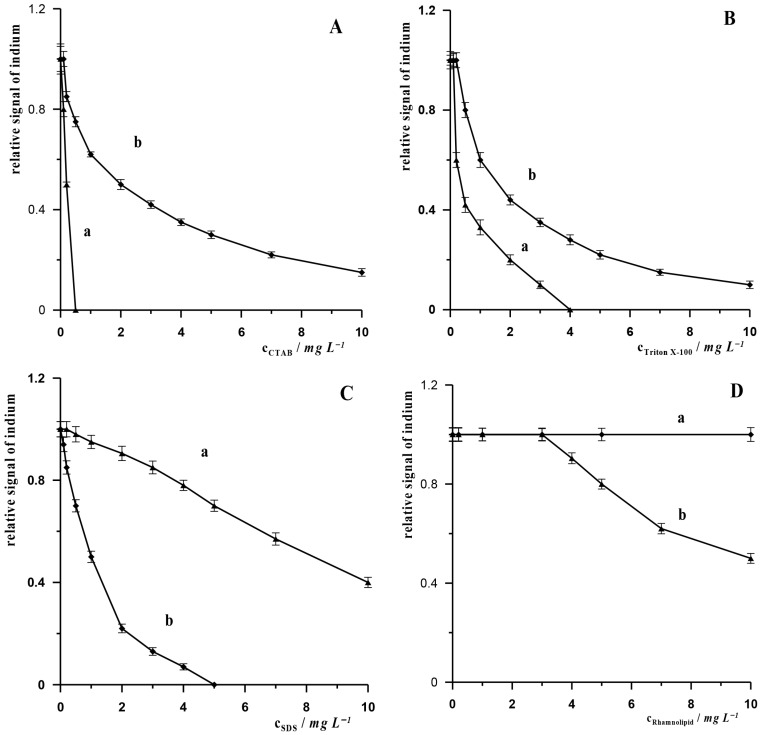

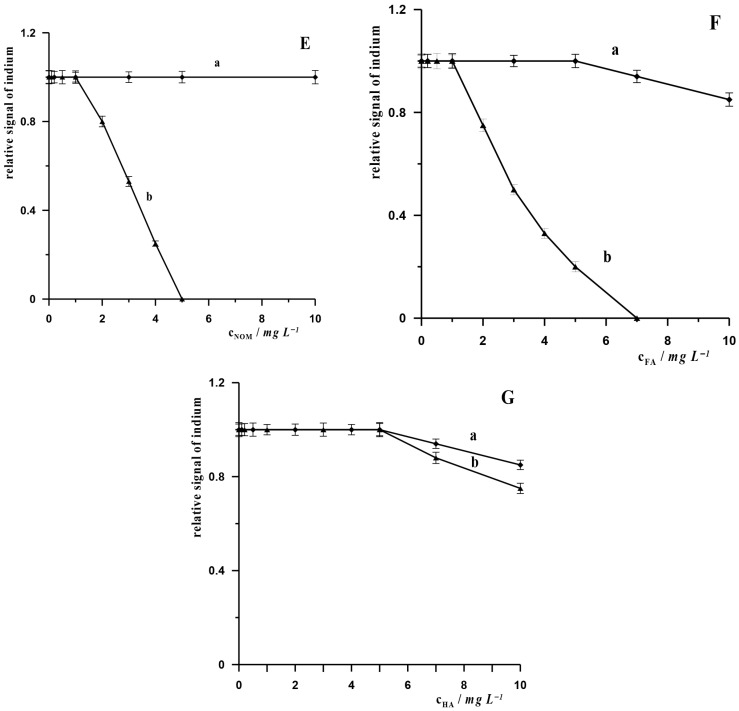
Relative signal of indium in the presence interferents: CTAB (**A**), Triton X-100 (**B**), SDS (**C**); Rhamnolipid (**D**), NOM (**E**), FA (**F**) and HA (**G**) for AdSV (curves a) and ASV (curves b) measurement. Concentration of In(III) 5 × 10^−8^ mol L^−1^. AdSV measurement conditions: activation potential and time −2.5 V and 45 s, accumulation potential and time −0.65 V and 10 s. ASV measurement conditions: activation potential and time −2.4 V and 20 s, accumulation potential and time −1.2 V and 20 s.

In the vast majority of procedures dedicated to the determination of In(III) in environmental samples, the influence of potential organic components present in the matrix of such samples has not been investigated. We drew attention to this problem in our previous work describing the AdSV procedure for the determination of In(III) using a bismuth film electrode. In that paper, we demonstrated that substances such as surfactants and humic substances can cause varying degrees of suppression of the indium signal, and we proposed a method to reduce these interferences by pre-mixing the sample containing such substances with XAD-7 resin [26]. The aim of the research in this work is to compare, for the first time, how surfactants and humic substances affect the analytical signal when we use the same working electrode and different measurement techniques, i.e., ASV and AdSV. We selected the following surfactants with different charges for the study: non-ionic surfactant—Triton X-100, anionic surfactant—sodium dodecyl sulphate (SDS) and cationic surfactant—cetyltrimethylammonium bromide (CTAB), biosurfactant—Rhamnolipid and humic substances: humic acid (HA), fulvic acid (FA) and natural organic matter (NOM). The measurements were carried out for synthetic samples containing a constant concentration of In(III) of 5 × 10^−8^ mol L^−1^ and various concentrations of the above-mentioned organic substances. The results obtained are presented in Figure 3A–G. These figures show how the indium signal changes in the presence of increasing concentrations of surfactants and humic substances: CTAB (Figure 3A), Triton X-100 (Figure 3B), SDS (Figure 3C), Rhamnolipid (Figure 3D) NOM (Figure 3E), FA (Figure 3F) and HA (Figure 3G). For each of the above-mentioned interferents, measurements were performed using both the ASV and AdSV techniques. As can be seen, depending on the method used, the tested interferents cause a reduction in the indium signal to varying degrees. For the AdSV technique, in the case of surfactants, cationic and neutral substances have a more suppressing effect on the indium signal compared to the ASV technique. Anionic substances, on the other hand, attenuate the signal more in the ASV technique than in the AdSV technique. The same relationship was observed in the case of humic substances, which attenuate the signal to a greater extent in the ASV technique than in the AdSV technique. This can be linked to their negatively charged functional groups. Humic substances are negatively charged primarily due to the dissociation of their acidic functional groups, such as carboxylic and phenolic groups. EDTA, which has a negative charge in solution, also causes greater interference when measurements are performed using the ASV technique. The presence of EDTA in the solution at a concentration of 1 × 10^−4^ mol L^−1^ causes a 60% reduction in the indium signal, while in measurements using the AdSV technique, the indium signal remains unchanged at an EDTA concentration of 2 × 10^−4^ mol L^−1^, and then gradually decreases, but the indium signal is still visible on the voltammetric diagram even at an EDTA concentration of 5 × 10^−3^ mol L^−1^. It can therefore be concluded that negatively charged organic substances suppress the indium signal in the ASV technique more than the signal recorded using the AdSV technique. In the case of neutral substances (Triton X-100), it can be observed that the indium signal is more attenuated in AdSV measurements, but the difference between the attenuation of signals recorded using ASV and AdSV techniques is insignificant. In the case of cationic substances, it is clear that the signal recorded using the AdSV technique is much more suppressed than in the case of the ASV technique.

A further conclusion from the analysis of the experiments is that, in general, surfactants (Figure 3A–C) attenuate the indium signal more than humic substances (Figure 3E–G), regardless of the measurement technique used.

### 2.7. Comparison of the Influence of Inorganic Ions on the Analytical Signal in the ASV and AdSV Procedures

In environmental samples, besides organic stuff, other potential interferers could be inorganic foreign ions. Their influence on the voltammetric signal of In(III) in procedures using bismuth electrodes has already been studied and described in the literature [26,32]. For our procedures using SBiµE, we investigated the effect of ions that could potentially have the most interfering effect, namely Ag(I), Cd(II), Cu(II), Pb(II), Sn(II), and Tl(I) on the analytical signal of In(III). Based on the results obtained, we assessed the extent to which the choice of ASV or AdSV measurement technique affects these interferences. In the case of the ASV procedure, the most interfering ions are Cd(II) and Ti(I), whose peak potentials are close to the peak potential of In(III). In the case of Pb(II), its oxidation peak appears at a more negative potential than the oxidation potential of In(III) and only affects the indium peak when it is present in at least a 5-fold excess. Similar interferences were observed in the ASV procedure previously described in the literature with a bismuth film electrode as the working electrode [32]. The other ions we tested, Ag(I), Cu(II), and Sn(II), do not affect the indium ASV signal even at their 100-fold excess. In the case of the AdSV procedure, at least 100-fold excesses of Ag(I), Cd(II), Cu(II), Pb(II), and Tl(I) ions do not affect the indium signal at all. Only Sn(II) causes attenuation of the indium signal at its 5-fold excess. These results are consistent with those observed previously when AdSV was used as the measurement procedure and a bismuth film electrode was used as the working electrode [26]. To summarise the effect of inorganic foreign ions, it can be concluded that the AdSV procedure is less sensitive to their influence compared to the ASV procedure and is recommended for the analysis of samples containing higher concentrations of inorganic foreign ions.

### 2.8. Seawater Analysis

**Figure 4:**
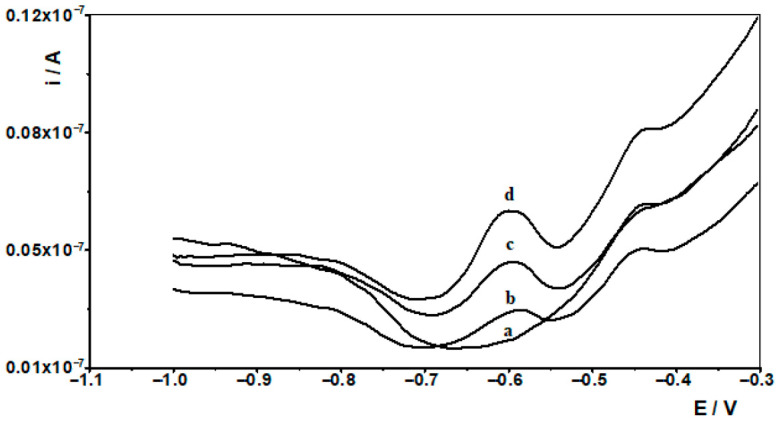
Differential pulse voltammograms obtained in the course of the In(III) ASV technique determination in Baltic sea. Baltic sea diluted ten times: (a). Baltic sea spiked with 1 × 10^−7^ mol L^−1^ In(III): (b) diluted ten times; (c) as (b) + 1 × 10^−8^ mol L^−1^ In(III); (d) as (b) + 2 × 10^−8^ mol L^−1^ In(III).

**Figure 5:**
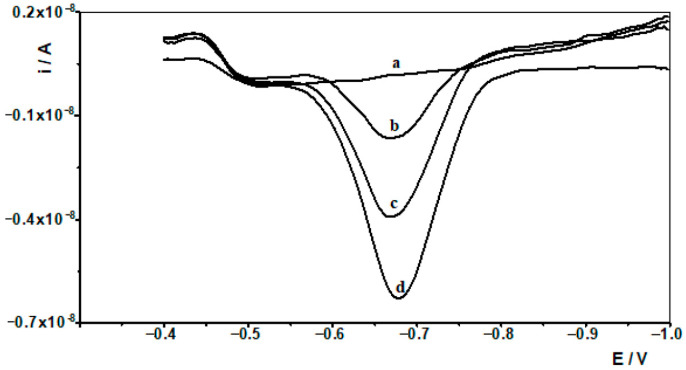
Differential pulse voltammograms obtained in the course of the In(III) AdSV technique determination in Baltic sea. Baltic sea diluted ten times: (a). Baltic sea spiked with 1 × 10^−7^ mol L^−1^ In(III): (b) diluted ten times; (c) as (b) + 1 × 10^−8^ mol L^−1^ In(III); (d) as (b) + 2 × 10^−8^ mol L^−1^ In(III).

Marine water pollution with metals is an anthropogenic problem originating from industrial and municipal wastewater, agricultural runoff and atmospheric pollution. These metals can lead to poisoning and disruption of vital functions in marine organisms, posing a threat to marine ecosystems and human health. While environmental waters such as rivers and lakes are often used as a matrix to confirm the applicability of developed voltammetric procedures, marine waters are less frequently chosen as a matrix. Therefore, in our study, we tested the applicability of both developed procedures by analysing the recovery of indium from marine water samples. The analysis was carried out using water from the Baltic Sea and Synthetic Sea Water purchased from Merck. The results obtained are presented in Table 1, and sample voltammograms for Baltic Sea are shown in Figure 4 and Figure 5.

## 3. Materials and Equipment

Suprapur or analytical grade chemicals were employed. Standard solutions of In(III) was prepared by diluting commercial standards containing 1000 mg L^−1^ from Fluka (Buchs, Switzerland). A solution of 1 × 10^−2^ mol L^−1^ of cupferron was prepared every day by dissolving 0.0155 g of the reagent in water in a 10 mL volumetric flask. A cupferron (benzenamine, *N*-hydroxy-*N*-nitroso-ammonium salt) was obtained from Merck (Darmstadt, Germany). An acetate buffer was prepared from Suprapur CH_3_COOH and NaOH obtained from Merck. The effect of surfactants was studied using the non-ionic surfactant Triton X-100, the anionic surfactant SDS and the cationic surfactant CTAB, all purchased from Fluka. The effects of humic acids HA (Aldrich, St. Louis, MO, USA), fulvic acids FA and natural organic matter NOM (both from the International Humic Substances Society) were also studied. Synthetic Sea Water was purchased from Merck (Darmstadt, Germany). All solutions were prepared with deionized water (resistivity > 18 MΩcm).

Voltammetric measurements were performed using a μAutolab PGSTAT 10 analyser (Utrecht, The Netherlands) and a personal computer with GPES software version 4.9 to control the system with a three-electrode configuration. The electrodes used were a solid bismuth microelectrode (SBiµE) with a diameter of 25 µm, a platinum wire auxiliary electrode, and an Ag/AgCl reference electrode. The solid bismuth microelectrode was manufactured by our research team and its structure and construction have been described in previous works [29,30]. The pH value of the buffer solution was measured using a digital pH meter with a glass combination electrode. The sonication of SBiµE was performed with an ultrasonic bath (Sonic-3, Polsonic, Warsaw, Poland).

## 4. Measurement Procedure

Each measurement day, the solid bismuth microelectrode was polished on 2500 silicon carbide paper (Matador company, Mörfelden-Walldorf, Germany). After that, the electrode was washed and sonicated for 30 s in an ultrasonic bath using deionized water to dislodge any remaining polishing material. All experiments were carried out without deaeration and at room temperature (20 ± 1 °C). Each measurement was repeated a minimum of five times, and the graphs represent the average calculated from these measurements.

### 4.1. ASV Measurement

Voltammetric measurements by anodic stripping voltammetry with differential pulse technique voltammogram registration were performed as follows: A 10 mL of solution containing 0.1 mol L^−1^ acetate buffer (pH = 3.0) and required concentration of the In(III) was placed in the electrochemical cell. Each voltammetric measurement began with electrochemical activation of the electrode surface by applying to it a potential of −2.4 V for 20 s. During this time, any bismuth oxide formed was reduced to its metallic form. The pre-accumulation stage was then started at the electrode applying a potential of −1.2 V for 20 s. During this time, the In(III) ions present in the solution are reduced to their metallic form and become concentrated at the SBiµE. Both of these steps were carried out in the solution stirred by a magnetic stirrer. After stopping the stirring followed by equilibration time of 5 s, the voltammograms were recorded in the anodic direction from −1.0 to −0.3 V with instrumental parameters of the differential pulse voltammetric measurement as follows: scan rate 40 mV s^−1^ and pulse height 50 mV. The voltammogram shows a peak associated with the oxidation of In(0) to In(III), the current value of which is proportional to the concentration of indium ions in the solution.

### 4.2. AdSV Measurement

Voltammetric measurements by adsorptive stripping voltammetry with differential pulse technique voltammogram registration were performed as follows. A 10 mL of solution containing 0.1 mol L^−1^ acetate buffer (pH = 3.0), 7 × 10^−5^ mol L^−1^ cupferron and required concentration of the In(III) was placed in the electrochemical cell. Each voltammetric measurement began with electrochemical activation of the electrode surface by applying a potential of −2.5 V to it for 45 s. The pre-accumulation stage was then started at the electrode applying a potential of −0.65 V for 10 s. During this time, the In(III)-cupferron complex present in the solution is adsorbed at the SBiµE surface. Both steps were carried out with the solution stirred by a magnetic stirrer. After stopping the stirring and after an equilibration time of 5 s, the voltammograms were recorded in the cathodic direction from −0.4 to −1.0 V with instrumental parameters of the differential pulse voltammetric measurement as follows: scan rate 40 mV s^−1^ and pulse height 50 mV. The voltammogram shows a peak associated with the reduction of In(III)-complex, the current value of which is proportional to the concentration of indium ions in the solution. Due to the fact that cupferron causes an increase in background current in the range of potentials close to the induction peak potential in all measurements performed using the AdSV technique, we applied background correction.

## 5. Conclusions

This paper showed that the solid bismuth microelectrode is a useful tool for the highly sensitive determination of In(III) by anodic stripping voltammetry and adsorptive stripping voltammetry. This mercury-free, non-toxic electrode based on solid bismuth, which does not require the introduction of bismuth ions into the solution of the analysed sample, therefore has excellent environmental advantages. Both developed procedures for the determination of In(III), ASV and AdSV, are characterised by high repeatability and reproducibility and a low detection limit, with the AdSV procedure having a detection limit five times lower than the ASV procedure. The influence of potential interferents, which are components of environmental water matrices such as surfactants, humic substances and EDTA, on the indium signal was compared. As noted, negatively charged organic compounds have a significantly more negative effect on the indium signal, causing it to decrease or disappear completely in measurements performed using the ASV technique. Neutral substances, and even more so acidic substances, cause greater interference in measurements performed using the AdSV technique compared to the ASV technique. Considering the fact that humic substances commonly present in environmental waters interfere less with the signal recorded by the AdSV technique compared to ASV and that the AdSV procedure allows for a lower detection limit than ASV in the analysis of environmental samples with low In(III) content, it is recommended to use a procedure based on the AdSV technique.

## Figures and Tables

**Table 1:** Examination of In(III) recovery from Baltic Sea and Synthetic Sea Water using BiSµE with ASV and AdSV techniques in keeping with optimised conditions.

Seawater	In(III) Added (nmol L^−1^)	ASV Technique			AdSV Technique		
		In(III) Found (nmol L^−1^)	In(III) Recovery (%)	RSD (%) *n* = 5	In(III) Found (nmol L^−1^)	In(III) Recovery (%)	RSD (%) *n* = 5
Baltic Sea	10.0	10.7	107	6.7	9.5	95	4.7
	20.0	18.8	94	5.8	19.4	97	4.2
Synthetic Sea Water	10.0	9.6	96	6.2	9.4	94	5.7
	20.0	21.2	106	5.6	19.0	95	5.1

## Data Availability

The original contributions presented in the study are included in the article, further inquiries can be directed to the corresponding author.
